# Editorial: Biomedical advances in ultrasound-mediated drug/molecule delivery

**DOI:** 10.3389/fphar.2022.974921

**Published:** 2022-08-11

**Authors:** Agata A. Exner, Jean-Michel Escoffre

**Affiliations:** ^1^ Case Western Reserve University, Cleveland, OH, United States; ^2^ UMR 1253, iBrain, Université de Tours, Inserm, Tours, France

**Keywords:** therapeutic ultrasound, biological barriers, drug delivery, pharmacokinetics, pharmacodynamics

Despite the increasing number of innovative drugs and the development of novel targeted methods, therapeutic advances remain modest for many prevalent and costly diseases including neurodegenerative disorders, cancers, and cardiovascular diseases among others. One of the major therapeutic hurdles is the presence of biological barriers in multiple organs (e.g., endothelial and epithelial barriers, plasma membrane, interstitial pressure, detoxification processes, etc.). While they sustain organ/tissue homeostasis in physiological conditions, these barriers substantially impede the delivery of a vast majority of therapeutic molecules (e.g., chemotherapeutics, antibiotics, nucleic acids, antibodies, etc.) in diseased tissues, thus reducing their bioavailability and therapeutic effect. This challenge narrows the landscape of usable therapeutic molecules and drastically influences the design of many therapeutic protocols. Therefore, crossing of biological barriers in drug studies is undoubtedly a source of major RD investments in academia and pharmaceutical industry.

For over 2 decades, therapeutic ultrasound (US) applications facilitating gene/drug delivery have been widely investigated, with some approaches being on the brink of reaching the bedside. Among these, using US-responsive particles injected systemically, e.g., microbubbles, to facilitate US-mediated, crossing of biological barriers has been shown to: 1) be applicable in a standardized and non-invasive fashion in laboratory animals and human subjects, and 2) render therapeutically-achievable drug/molecule biodistribution, supporting the clinical translatability of this modality. While these advances foresee a “blue sky” in the field, like in many medical specialties, the translation gap remains challenging to evaluate. One may ask - to *what extent is a successful pre-clinical study predictive of the outcome of its clinical counterpart?* Before devising a clinical study, it is essential to boost chances of clinical success by conducting impactful *in-vitro* and preclinical studies that can inform clinical trial design and enable technology translation.

This Research Topic promotes four original contributions in the fields of bactericidal therapy, alopecia, cancer and gene therapy from 27 authors: three from Asia and one from Europe. All findings reported in the Research Topic had 4,912 views on 1 June 2022 (Source: Frontiers in Pharmacology).

Bacterial infection and inflammation are strongly involved in the pathogenesis of androgenic alopecia (Sakr et al., 2013). Minoxidil and lysozyme are both potent bioactive agents that were evidenced to display bactericide properties and to promote the enhancement of hair follicle growth (Springer et al., 2003; Yamasaki and Gallo, 2008); their codelivery requires a novel encapsulation formulation. In this issue, Liao and others report the design and the *in-vitro* and *in-vivo* validation of a new type of minoxidil-coated lyzozyme-shelled microbubble (MB) for inhibiting bacterial infections and for enhancing hair follicle growth (Liao et al.). The authors showed an increased penetration of minoxidil and lysozyme into the skin and an enhancement of hair growth compared to minoxidil treatment alone.

The exploitation of a clinical US scanner and clinically-approved microbubbles for enhanced drug delivery is increasing interest to facilitate the clinical translation of US-mediated drug delivery methods (Lammertink et al., 2015). Here, two articles describe that clinical US scanner with their respective probes (Philips iU22 with S5-1 probe; Canon Aplio XG SSA-790 with PLT-704SBT probe) and clinically-approved MBs (SonoVue; Sonazoid) can safely and effectively enhance the drug concentration (bleomycin; pirarubicin) *in-vitro* (De Maar et al.) and *in-vivo* (Sasaki et al.)*.*

Recently, nanobubbles (NBs) have attracted attention as an alternative option to MBs for US-mediated drug and gene delivery (Exner and Kolios, 2021). In this Research Topic, Kida et al. report the influence of NB size distribution on US-mediated plasmid DNA (pDNA) and messenger RNA (mRNA) delivery. They demonstrated that NBs with size greater than 200 nm resulted in higher transfection efficiency *in vitro*. We look forward to consulting future results on the underlying mechanism for this method and on its efficacy to deliver therapeutic pDNA and mRNA.

In conclusion, this Research Topic of relevant contributions shedding the light on the biomedical advances in US-mediated drug delivery. Altogether these results show the drive 1) to develop this approach for new clinical applications (bacterial infections, gene therapy) and 2) to transfer it to the clinic. But they also highlight the need to develop new US-sensitive drug carriers (drug-loaded MBs, NBs) in order to answer clinical needs.
